# Common and novel metabolic pathways related ESTs were upregulated in three date palm cultivars to ameliorate drought stress

**DOI:** 10.1038/s41598-022-19399-8

**Published:** 2022-09-02

**Authors:** Mohammed Refdan Alhajhoj, Muhammad Munir, Balakrishnan Sudhakar, Hassan Muzzamil Ali-Dinar, Zafar Iqbal

**Affiliations:** 1 Department of Arid Land Agriculture, College of Agriculture and Food Sciences, King Faisal University, PO Box 31982, Al-Ahsa, Saudi Arabia; 2 Date Palm Research Center of Excellence, King Faisal University, PO Box 31982, Al-Ahsa, Saudi Arabia; 3 Central Laboratories, King Faisal University, PO Box 31982, Al-Ahsa, Saudi Arabia

**Keywords:** Biotechnology, Molecular biology, Physiology

## Abstract

Date palm is an important staple crop in Saudi Arabia, and about 400 different date palm cultivars grown here, only 50–60 of them are used commercially. The most popular and commercially consumed cultivars of these are Khalas, Reziz, and Sheshi, which are also widely cultivated across the country. Date palm is high water-demanding crop in oasis agriculture, with an inherent ability to tolerate drought stress. However, the mechanisms by which it tolerates drought stress, especially at the transcriptomic level, are still elusive. This study appraised the physiological and molecular response of three commercial date palm cultivars Khalas, Reziz, and Sheshi at two different field capacities (FC; 100% and 25%) levels. At 25% FC (drought stress), leaf relative water content, chlorophyll, photosynthesis, stomatal conductance, and transpiration were significantly reduced. However, leaf intercellular CO_2_ concentration and water use efficiency increased under drought stress. In comparison to cvs. Khalas and Reziz, date palm cv. Sheshi showed less tolerance to drought stress. A total of 1118 drought-responsive expressed sequence tags (ESTs) were sequenced, 345 from Khalas, 391 from Reziz, and 382 from Sheshi and subjected to functional characterization, gene ontology classification, KEGG pathways elucidation, and enzyme codes dissemination. Three date palm cultivars deployed a multivariate approach to ameliorate drought stress by leveraging common and indigenous molecular, cellular, biological, structural, transcriptional and reproductive mechanisms. Approximately 50% of the annotated ESTs were related to photosynthesis regulation, photosynthetic structure, signal transduction, auxin biosynthesis, osmoregulation, stomatal conductance, protein synthesis/turnover, active transport of solutes, and cell structure modulation. Along with the annotated ESTs, ca. 45% of ESTs were novel. Conclusively, the study provides novel clues and opens the myriads of genetic resources to understand the fine-tuned drought amelioration mechanisms in date palm.

## Introduction

Date palm (*Phoenix dactylifera* L., family *Arecaceae*) is a monocot, dioecious, perennial woody fruit tree with a genome size of ca 650 Mbp^1–3^. Nonetheless, a recent study reported that the date palm genome is 18% larger and more contiguous than earlier reports^3^. It is an ancient plant commodity in the Arabian Peninsula and possibly originated from Iraq. Date palm is cultivated in both the Old World (Africa, Asia, and Europe) and the New World (American continent), and the cultivation area has been stretched from the East Indus Valley to the West Atlantic Ocean^4^. Total worldwide dates production is ca. 9.61 million tonnes, and the area under cultivation is 1.25 million hectares^5^. The Kingdom of Saudi Arabia (KSA) is the epicenter of date palm diversity with approximately 400 cultivated cultivars and ranked fourth in date production with 1.54 million tonnes from ca. 152 hundred thousand ha cultivated area^5,6^. Among 400 cultivated cultivars, Khalas, Reziz and Sheshi are among the most popular cultivars based on consumer preference, particularly in the Eastern province of KSA^7^.

Many abiotic factors are responsible for a severe reduction in yield and life span of date palm cultivars in the Arabian Peninsula, especially in KSA^8^. Additionally, such abiotic stressors exacerbate the spread of pests and diseases^9^. According to estimates, certain abiotic stresses will cause up to a 50% reduction in the average crop yield by the mid of this century^10^. Although drought is a global phenomenon that could affect around 50% of arable land, the situation in the Arabian Peninsula is more alarming. Drought impairs plant growth, photosynthetic rate, nutrient and water relations, assimilate partitioning, ultimately resulting in significant crop yield reduction^11^. The development and screening of abiotic stress-tolerant cultivars of dryland crops become a high priority to confront the aggravating scenario. Nevertheless, no sophisticated breeding program has opted for date palm, but different cultivars were randomly selected based on yield and quality^12^. Therefore, a high degree of heterogeneity in date palm germplasm is likely to be present with respect to drought. All plants, including date palm, have developed fine-tuned mechanisms to respond to drought stress at various morphological, physiological, biochemical and molecular levels.

Drought is becoming one of the most prominent environmental challenges as a result of climate change, limiting plants' physiological characteristics^13^. When plants are subjected to drought, their photosynthetic machinery is altered, which can cause changes in photosynthate source/sink relationships and plant development^14^. Plant roots absorb water in order to utilize it for photosynthesis and biomass production. Water is consumed in a small amount, with most of it being returned to the environment via transpiration. Stomata are important for synchronizing photosynthetic and transpiration processes. Stomata take CO_2_ gas from the environment and return water through stomatal conductance. One of the most effective mechanisms that plants use to control the amount of water that evaporates into the atmosphere during drought is stomatal closure^15^. Drought causes an over-accumulation of reactive oxygen species, decreasing photosynthetic activity by inhibiting and degrading chlorophyll synthesis^16,17^. The relative water content of the leaves reduces during drought, resulting in the reduction of stomatal conductance, photosynthetic rate, and transpiration. In date palm cvs. Sheshi^18^ and Khalas^19^, water stress decreased chlorophyll, photosynthetic rate, stomatal conductance, and transpiration while increasing water use efficiency (WUE). Date palm cultivars showed an increase in WUE while decreasing gas exchange characteristics under drought stress in vitro conditions^20^.

Drought tolerant plants have certain genetic traits that help them withstand for a longer time during drought stress. Plant species can undergo various morphological, physiological, biochemical, metabolical, and molecular changes that enable them to survive under drought stress and re-establish normal cell function after stress cessation^21^. At molecular levels, generally, two groups of such genes are known; the first group protects directly from drought and include osmotin, chaperones, water channel proteins, late embryogenesis abundant proteins, osmolyte biosynthesis, mRNA binding proteins, and sugar and proline transporter^22^. While the second regulates a diverse array of drought-responsive regulatory genes and include transcription factors, phosphatases, signaling molecules (calmodulin-binding proteins), and protein kinases^22,23^. Plants regulate a plethora of genes at the molecular level to alleviate the stress. Some recent advances have been made to understand the complex cascade of gene expression during osmotic stress, particularly in abscisic acid (ABA)-dependent and ABA-independent signaling pathways. ABA is an important key stress-signaling hormone in plants. In ABA-dependent pathway, hyper-production of ABA is achieved to mitigate the drought. In turn, the ABA production triggers underlying cascade pathways such as the production of reactive oxygen species (ROS), elevation of cytosolic calcium levels, and activation of ion channels to achieve the stomatal closure. In ABA-dependent pathway, ABA-responsive element (ABRE) and ABRE-binding transcription factors (ABFs) play a pivotal role. Likewise, in ABA-independent pathway, cis-element, dehydration-responsive element/C-repeat (DRE/CRT) and DRE-binding protein 2 (DREB2) play important roles. The detailed role of key transcription factors: DREB1/cis-element binding factors (CBFs), DREB/DRE, AREB/ABFs, DREB2A, and DREB1/CBF3, in transcriptional regulation of ABA-dependent and ABA-independent gene expression have been extensively reviewed^24,25^. Besides, many other genes such as late embryogenesis abundant, photosystem, ATP synthesis, monooxygenases and DREB/CBF, are up-regulated to neutralize the adverse effects of stress^26^.

Plants have enormous biological and genetic diversity, so they deploy different transcriptome/proteomic approaches to offset the drought stress. However, expression levels and underlying molecular mechanisms could vary in various plant species. Therefore, it is of prime importance to investigate and unravel the drought-responsive genes in different cultivars of the same plant species to understand their stress adaptation role. Unfortunately, apart from socio-economic values and global cultivation, date palm is among those whose genomes are less studied and characterized. Until now, just a few studies are available where stress-responsive genes in date palm were studied at the molecular level^27–30^. As the date palm genome is the least studied genome; consequently, minimal information is available regarding its genome annotation and functional characterization.

The transcriptomic analysis enables deciphering how a crop's intricate molecular machinery responds to abiotic stress. The suppression subtraction hybridization (SSH) technique has a substantial advantage in investigating the gene regulations in response to particular/induced stress. Additionally, it serves as an alternative to microarray in transcript profiling of novel genes^31^. The PCR-Select cDNA Subtraction technique is a unique method for selectively amplifying differentially expressed sequences, which overcomes the technical limitations of traditional subtraction methods^32^.

In the study outlined here, the response of three date palm cvs. Khalas, Reziz and Sheshi, to drought stress at the physiological and molecular level was investigated. The results demonstrated that the leaf physiological characteristics were negatively affected under drought conditions, and the nexus of drought-responsive gene regulation shared several similitudes and dissimilitude in these date palm cultivars. The significance of the findings is discussed in more detail.

## Results

### Changes in physiological variables under water stress

**Figure 1:**
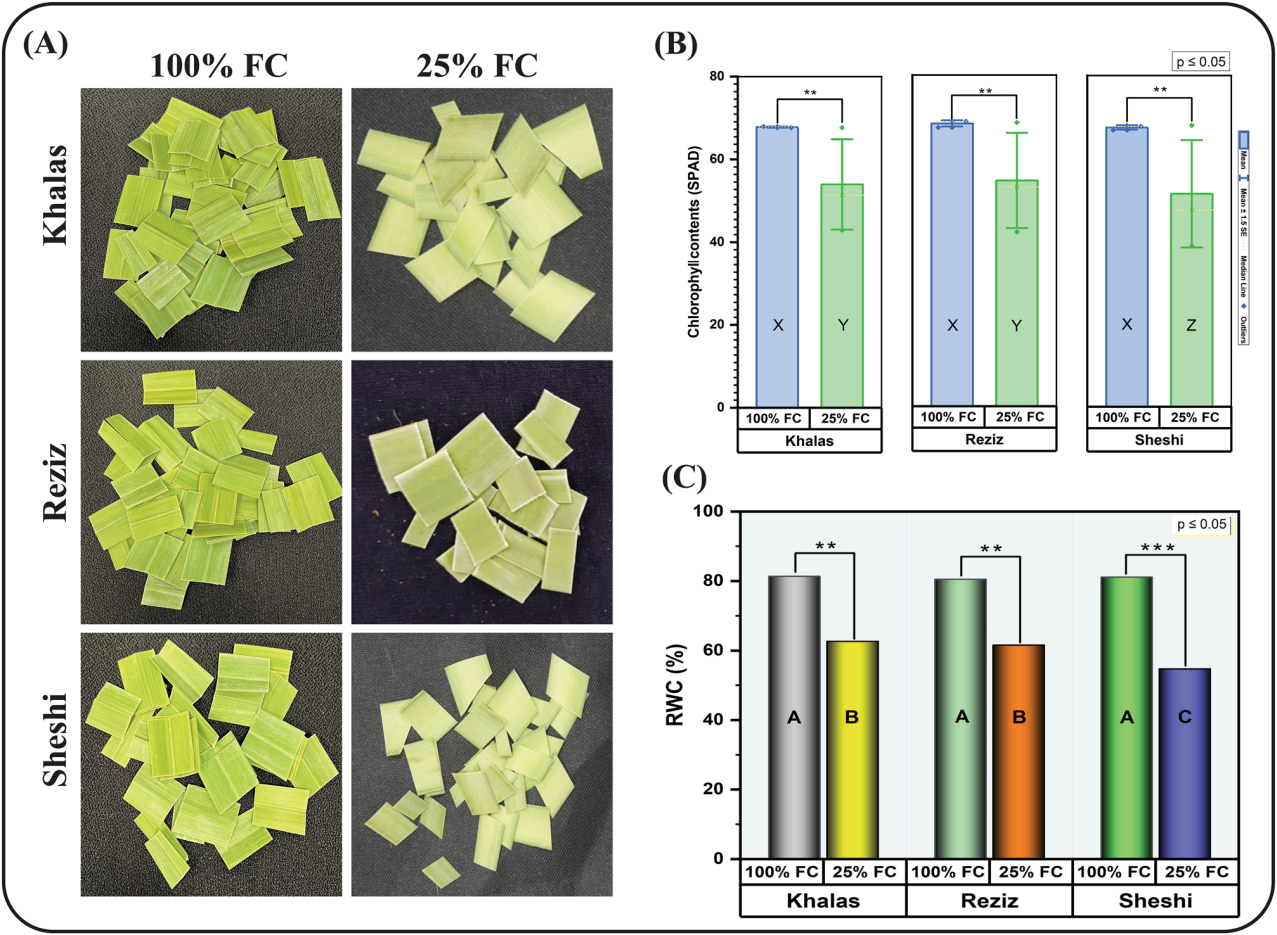
Phenotype exhibited by date palm leaves (**A**), chlorophyll contents (**B**), and relative water contents (**C**) at 100% and 25% field capacity. Bar graph showing different letters are significantly different at *p* ≤ 0.05, and the asterisk represents the significance of the level of probability. The data presented here represents the chlorophyll content and relative water content recorded after 90 days of drought induction.

All three date palm cultivars (Khalas, Reziz, and Sheshi) exhibited a comparable level of phenotypes (Fig. 1A). At 100% FC, these plants had significantly higher physiological parameters than plants at 25% FC (water stress). The chlorophyll contents (Fig. 1B) and RWC (Fig. 1C) were decreased after 90 days of water stress, whereas photosynthesis, stomatal conductance, and transpiration (Table 1) were also decreased from 0 to 45 and 90 days of water stress. However, intercellular CO_2_ concentration was increased with the increase in water stress (Table 1). The WUE values were not significantly affected after 45 days of water stress; however, after 90 days of water stress, the WUE was significantly higher in cv. Khalas (Table 1). Comparing date palm cultivars after 90 days of water stress, cvs. Khalas and Reziz showed higher chlorophyll content and RWC than the cv. Sheshi at 25% FC. The values regarding photosynthesis, stomatal conductance, and transpiration attributes recorded after 45 and 90 days of water stress were higher in cvs. Khalas and Reziz. These attributes were lowest in date palm cv. Sheshi after same time intervals. However, due to water stress, the intercellular CO_2_ concentration was significantly higher in cv. Sheshi compared to the other two cultivars after 45 and 90 days of stress.

**Table 1:** Photosynthesis (*Pn*), stomatal conductance (*gs*), transpiration (*E*), intercellular CO_2_ concentration (*Ci*), and water use efficient (WUE) of date palm cvs. Khalas, Reziz, and Sheshi at 100 and 25% field capacity.

Date palm cultivars	*Pn* (µmol m^-2^ s^-1^)			*g*_*S*_ (mmol m^-2^ s^-1^)			*E* (mmol m^-2^ s^-1^)			*Ci* (µmol mol^-1^)			WUE		
	Sampling time (days)			Sampling time (days)			Sampling time (days)			Sampling time (days)			Sampling time (days)		
	0	45	90	0	45	90	0	45	90	0	45	90	0	45	90
Khalas_(100% FC)_	10.04^a^	10.10^a^	10.29^a^	56.67^a^	57.30^a^	57.70^a^	1.48^a^	1.49^a^	1.51^a^	200.50^a^	205.57^d^	204.89^c^	6.80^a^	6.78^a^	6.81^b^
Khalas_(25% FC)_	10.06^a^	7.09^b^	5.67^b^	57.97^a^	41.47^b^	31.95^b^	1.51^a^	1.01^b^	0.73^b^	202.81^a^	247.11^c^	296.71^b^	6.66^ab^	7.05^a^	7.77^a^
Reziz_(100% FC)_	10.01^a^	10.03^a^	10.15^a^	57.10^a^	57.40^a^	56.63^a^	1.49^a^	1.51^a^	1.50^a^	205.09^a^	202.62^d^	206.25^c^	6.70^ab^	6.66^a^	6.76^b^
Reziz_(25% FC)_	9.96^a^	6.88^b^	4.99^c^	56.83^a^	40.31^b^	31.60^b^	1.50^a^	1.02^b^	0.72^b^	203.37^a^	255.68^b^	301.01^b^	6.64^b^	6.76^a^	6.91^b^
Sheshi_(100% FC)_	10.03^a^	10.05^a^	10.11^a^	57.90^a^	57.57^a^	56.90^a^	1.49^a^	1.47^a^	1.50^a^	203.22^a^	201.48^d^	205.16^c^	6.73^ab^	6.84^a^	6.72^b^
Sheshi_(25% FC)_	10.04^a^	6.16^c^	3.99^d^	57.23^a^	33.82^c^	24.95^c^	1.49^a^	0.87^c^	0.64^c^	202.09^a^	284.66^a^	348.05^a^	6.75^ab^	7.06^a^	6.24^b^

Different letters within each column indicate significant mean difference at *p* ≤ 0.05. The statistical analysis was based on a completely randomized design (CRD). Each data are the mean of three independent replicates. Duncan’s Multiple Range Test (DMRT) was applied to determine the least significance difference (LSD) between treatment means.

### Sequencing of the drought-responsive ESTs

Functional genomics approaches, such as EST, have been used extensively to understand the stress-response mechanism in plants.Three independent subtractive cDNA libraries of date palm cvs. Khalas, Reziz, and Sheshi, were constructed from the middle leaf tissues of drought-exposed date palm plants. A total of 2733 drought-responsive ESTs from three date palm cultivars were isolated, cloned, and sequenced. After trimming the vector sequences, eliminating low quality, duplicated, and short read-length sequences, collectively 1118 high-quality ESTs, 345 from Khalas, 391 from Reziz, and 382 from Sheshi, were subjected to the analysis. A total of 1107 ESTs were submitted to the databank after precluding those ESTs having a length of fewer than 100 nucleotides (due to databank requirements), comprising 344 from Khalas (Bioproject PRJDB11270; accession # HX998187-HX998530), 387 from Reziz (Bioproject PRJDB11271 accession # HX998531-HX998917), and 376 from Sheshi (Bioproject PRJDB11272; accession # HX998918-HX999293), and can be accessed online (https://www.ncbi.nlm.nih.gov/bioproject/).

### Functional annotation and characterization of all ESTs

**Figure 2:**
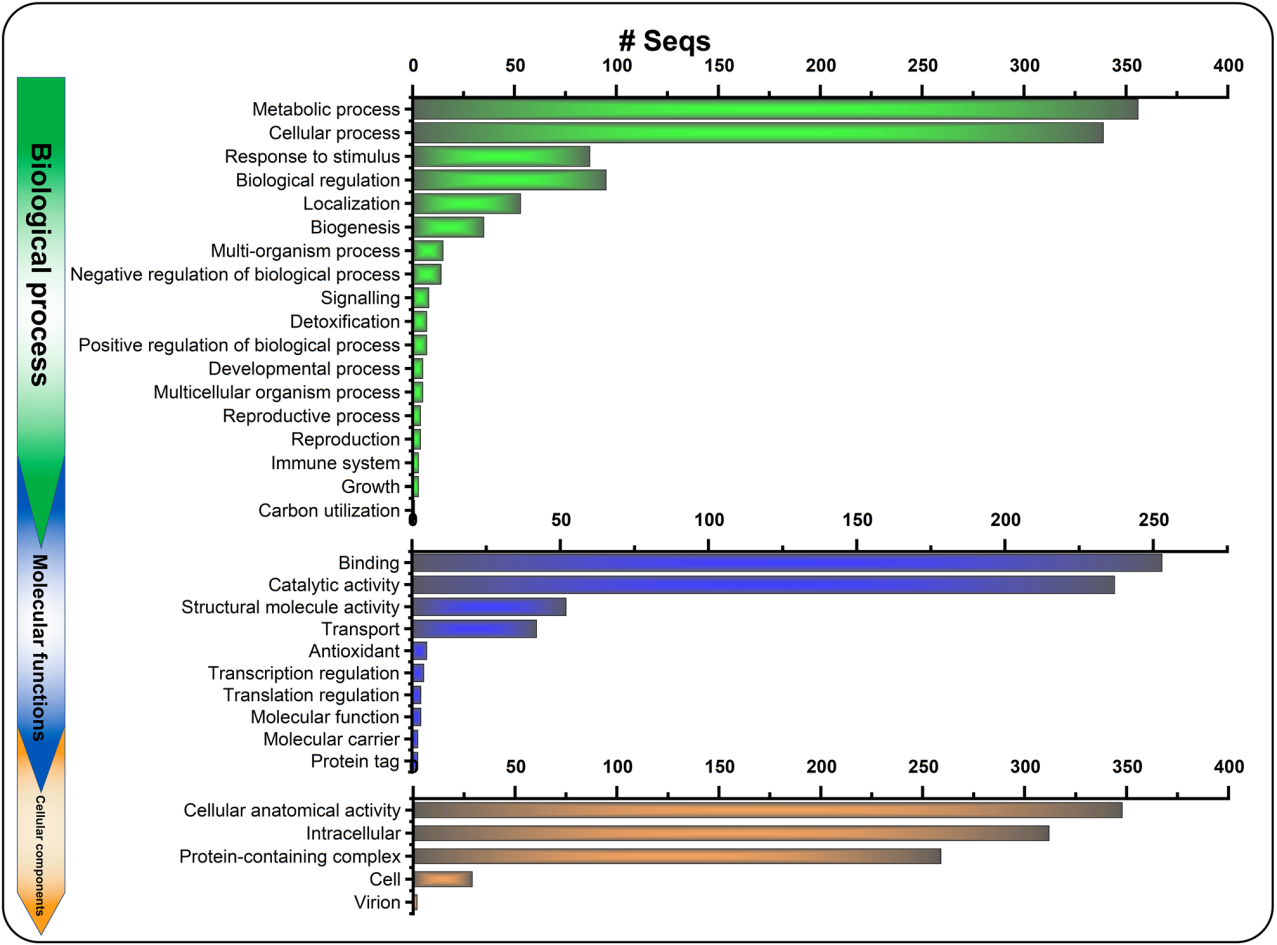
The gene ontology (GO) of all drought-responsive ESTs in three date palm cultivars. The y-axis showed activities to which ESTs belong biological process, molecular functions, and cellular components, and the x-axis represents their number of ESTs.

Initially, all the EST sequences obtained from three cultivars were BLASTx searched (https://blast.ncbi.nlm.nih.gov/). These findings indicated that ESTs were related to metabolic and cellular processes, biological regulations, biogenesis, binding and catalytic proteins, photosynthesis, integral cell membrane components, oxidative phosphorylation, translation, transcription, and immune systems. Subsequently, all the isolated ESTs were subjected to direct gene annotation (GO) for assigning biological processes (BP), molecular functions (MF), and cellular components (CC) functions. The BP results revealed that the highest number of ESTs (356 [31.85%] of 1118) were found related to metabolic process, followed by cellular process (339 [30.32%] of 1118), biological regulation (95 [8.5%] of 1118), and response to stimulus (87 [7.80%] of 1118) (Fig. 2). The MF analysis found that the highest number of upregulated ESTs belonged to the binding function (253 [22.30%] of 1118), followed by catalytic activity (237 [21.20%] of 1118), structural molecule activity (52 [4.65%] of 1118), and transport (42 [3.76%] of 1118) (Fig. 2). The CC analysis mapped that the highest number of upregulated ESTs were associated with cellular and anatomical activity (348 [31.13%] of 1118), followed by intracellular activity (312 [27.91%] of 1118), and protein-containing complex (259 [23.17%] of 1118) (Fig. 2).

The direct GO count analysis based on high-score non-redundant BLASTx homology was also carried out against various plant species (Figure S1). The direct GO count BP analysis exhibited that the highest number of upregulated ESTs (172 [15.40%] of 1118) were involved directly or indirectly in the photosynthesis process (including light harvesting, photosynthetic electron transport in PSI and PSII, and photorespiration), followed by the oxidation–reduction process (87 [7.80%]), protein-chromaphore linkage (63 [5.64%]), translation (44 [3.94%]), and ATP synthesis (~ 18 [1.61%]) (Figure S1). The direct GO count CC analysis showed that 306 (27.37% of 1118) upregulated ESTs were chloroplast related (including chloroplast, chloroplast envelope, stroma, and thylakoid), 291 (26.02%] of 1118) were membrane components related (including integral component of membrane, thylakoid membrane, and plasma membrane), and 221 (19.77%) were photosynthesis related (including PSI, PSII, PSII oxygen evolving complex, and PSI reaction center). According to the direct GO count MF analysis, the highest number of ESTs (116 [10.38% of 1118) were related to different enzymatic activities (including rubisco, monooxygenase, hydrolase, oxidoreductase, and ATP synthase), 67 (6.0% of 1118) upregulated ESTs were related to metal ion binding (including copper and zinc ion binding), 64 (5.72%] were related to chlorophyll binding, and 52 (3.75%) ESTs were related to ATP binding (Figure S1).

The organism distribution of all ESTs revealed that the highest number of ESTs (ca. 375) did not reveal any organism-specific similarity. Nonetheless, the highest numbers of ESTs (ca. 22) were found to be similar to *Chlaymydomonas reinhardti*, followed by *Malus domestica* (13 ESTs), *Quercus suber* (13 ESTs), *Coffea canephora* (13 ESTs), and *Phaseolus vulgaris* (12 ESTs). However, just a few ESTs (ca. 10 ESTs) showed a top hit with date palm (Figure S3).

### Cultivar–wise functional annotation

**Figure 3:**
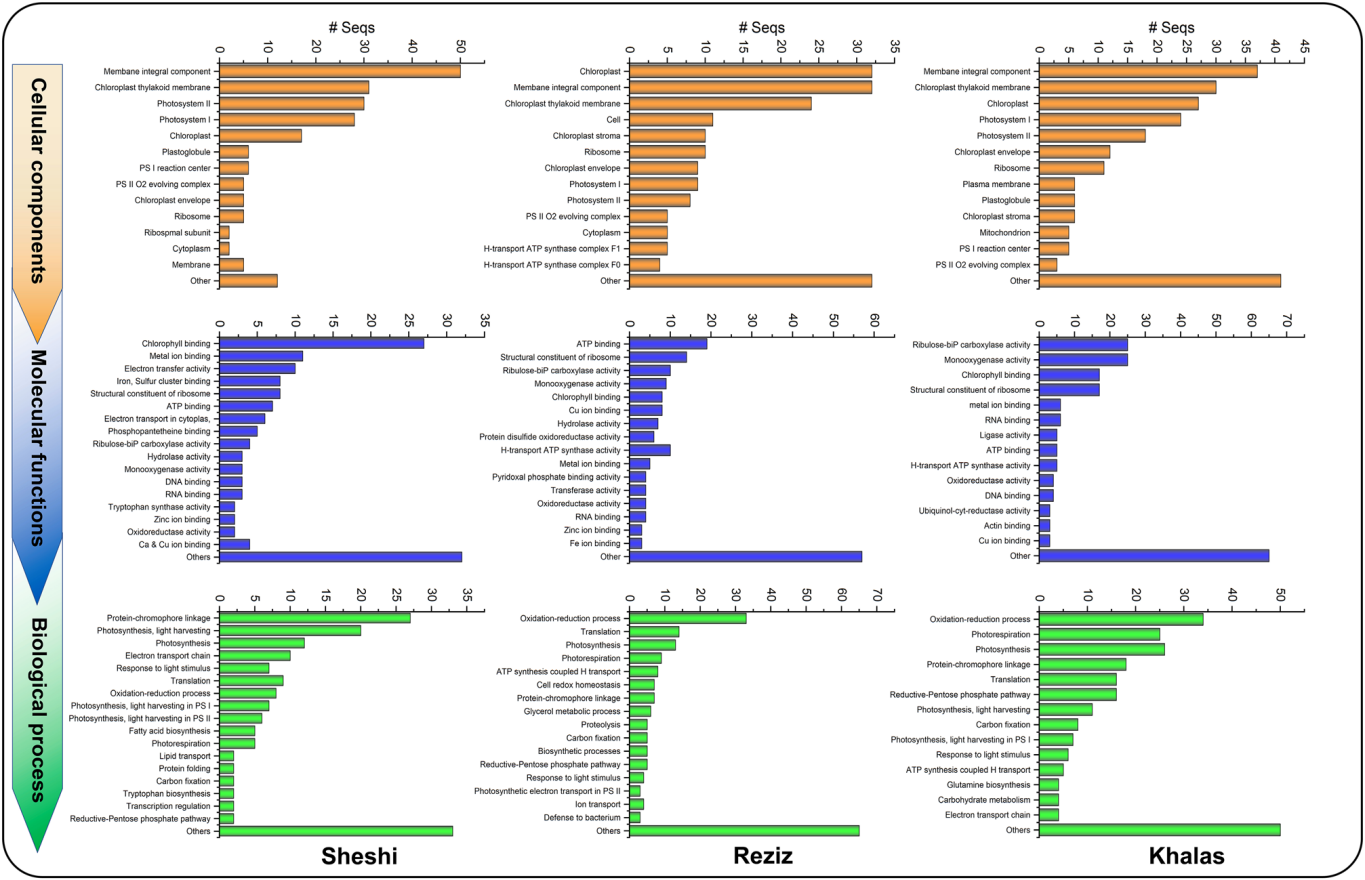
Relative comparative gene ontology (GO) of drought-responsive ESTs in three date palm cultivars. GO terms were organized contingent on p values ≤ 0.05. The x-axis showed activities to which ESTs belong to cellular components, molecular functions, and biological.

ESTs isolated from each cultivar were subjected to functional annotation, and their cellular components (CC), molecular functions (MF), and biological process (BP) were inferred. The CC analysis revealed that the highest number of ESTs related to integral membrane components were expressed in Sheshi (50 ESTs), while both Khalas and Reziz expressed an equal number of ESTs (35) (Fig. 3). Coherently, about 30 ESTs encoding chloroplast thylakoid membrane were found in each cultivar. Reziz expressed the most ESTs (32) encoding chloroplast genes, followed by Khalas (26 ESTs) and Sheshi (18 ESTs) (Fig. 3). Interestingly, Khalas had the highest number of ESTs pertaining to chloroplast envelope (12 ESTs), followed by Reziz (8 ESTs) and Sheshi (5 ESTs), respectively. The highest number of ESTs related to photosystem I and II were expressed in Sheshi, 28 and 29, respectively, followed by Khalas (24 and 15 ESTs) and Reziz (9 and 8 ESTs). Mutually, a total of 25 ESTs encoding ribosomes related proteins were expressed, 10 ESTs in each Khalas and Reziz, while just 5 ESTs in Sheshi (Fig. 3).

The MF analysis identified 25 ESTs encoding Ribulose-1,5-bisphosphate carboxylase (Rubisco) in Khalas, 10 ESTs in Reziz, and just 4 ESTs in Sheshi (Fig. 3). The second most abundant ESTs mapped were those encoding monooxygenase activity, accounting for 25, 8, and 3 ESTs in Khalas, Reziz and Sheshi, respectively. Another large set of ESTs expressed in the date palm cultivars, 15 ESTs in Khalas, 8 in Reziz and 27 in Sheshi, were related to chlorophyll-binding proteins (Fig. 3). Metallothioneins (metals ion binding proteins [MIB]) were another substantial group of proteins expressed in date palm cultivars. Different MIB including copper ion binding (CIB; ca. 15 ESTs), ferric ion binding, calcium ion binding, and zinc ion binding (ZIB; ca. 10 ESTs) proteins were expressed; the highest number of MIB ESTs were expressed in Reziz followed by Khalas and Sheshi (Fig. 3). The other major groups of ESTs expressed in all three cultivars were belonging to structural constituents of ribosomes, ATP binding proteins, genome (DNA and RNA)-binding proteins and oxidation–reduction related proteins (Fig. 3).

The BP analysis exhibited that the majority of drought-responsive ESTs were related to the oxidation–reduction process; 34 ESTs were expressed in Khalas, 33 in Reziz, and 8 in Sheshi (Fig. 3). In total, 76 photosynthesis-related ESTs were upregulated in all three cultivars (52 related to photosynthesis and 24 to light harvestation PS I and II). Photosynthesis related ESTs upregulated in Khalas were 26, while 13 were upregulated in each, Reziz and Sheshi. Another area where 38 ESTs were expressed mutually was photorespiration; 25 ESTs for Khalas, 8 ESTs for Reziz, and 5 ESTs for Sheshi (Fig. 3). Many ESTs encoding chromaphore linkage proteins were expressed; Sheshi had the highest number (24 ESTs), followed by Khalas and Reziz with 17 and 7 ESTs, respectively. Another substantial number of ESTs related to the translation process was expressed in all three cultivars; 16 ESTs were expressed in Khalas, 14 in Reziz, and 8 in Sheshi.

Khalas expressed the most ESTs related to the pentose phosphate pathway (16 ESTs), followed by Reziz (5 ESTs) and Sheshi (2 ESTs) (Fig. 3).

Besides the common set of ESTs; the indigenously unique ESTs enriched in CC, MF and BP were upregulated in all three cultivars. In Khalas, the unique enriched ESTs involved in CC were related to plastoglobule, Photosystem-I (PS-I) reaction centre, and PS-II-O_2_ evolving complex. In Reziz, H-ion transport, ATP synthase complex F0 and F1. Similarly, ligase and ubiquinol-cytochrome-reductase were unique ESTs involved in MF in Khalas. Whereas, protein di-sulphide reductase, pyridoxal-phosphate binding, and hydrolase were found in Sheshi, tryptophan synthase, hydrolase, and phosphopantothiene binding were found in Reziz (Fig. 3). Unique ESTs related to BPs in Khalas included carbohydrate metabolism and glutamine biosynthesis; in Reziz included proteolysis, defense to bacteria, and glycerol metabolism; and in Sheshi included tryptophan biosynthesis, lipid transport, and fatty acid biosynthesis (Fig. 3). Apart from these common and novel ESTs, the majority of ESTs were found to be novel, and their functions (CC, MF, and BP) could not be ascribed; consequently, all of these ESTs were labelled as “others” in Fig. 3.

processes, and the y-axis represents their number of ESTs.

### KEGG pathways and enzyme code distribution analysis

Following GO annotation, assigning KO and enzyme code (EC) to the ESTs, the KEGG pathways were elucidated, and the results demonstrated that drought-responsive ESTs were associated with over 50 pathways (Table S1). Approximately 400 ESTs (35% of 1118) were categorized into different metabolic pathways, including biosynthesis of antibiotics (65 ESTs [17% of 400] related to 19 different enzymes), carbon fixation in photosynthesis (55 ESTs [14% of 400] related to 12 different enzymes), glyoxalate and dicarboxylate metabolism (51 ESTs [12.75% of 400] related to 4 different enzymes), Arginine biosynthesis (15 ESTs), Alanine, aspartate, and glutamate metabolism (15 ESTs), Nitrogen metabolism (13 ESTs), Pentose phosphate pathway (10 ESTs related to 6 different enzymes), glycolysis/gluconeogenesis (9 ESTs related to 6 different enzymes), Purine metabolism (9 ESTs related to 4 different enzymes), Thiamine metabolism (8 ESTs), biosynthesis of phenylalanine, tyrosine and tryptophan (8 ESTs) and fructose and mannose metabolism (Figure S2). The other pathways were related to fatty acid, alkaloid, and phenylpropanoid biosynthesis. Since we only include date palm leaves in the study, so these results explicitly focus on the gene expression profile of leaves metabolic pathways.

**Figure 4:**
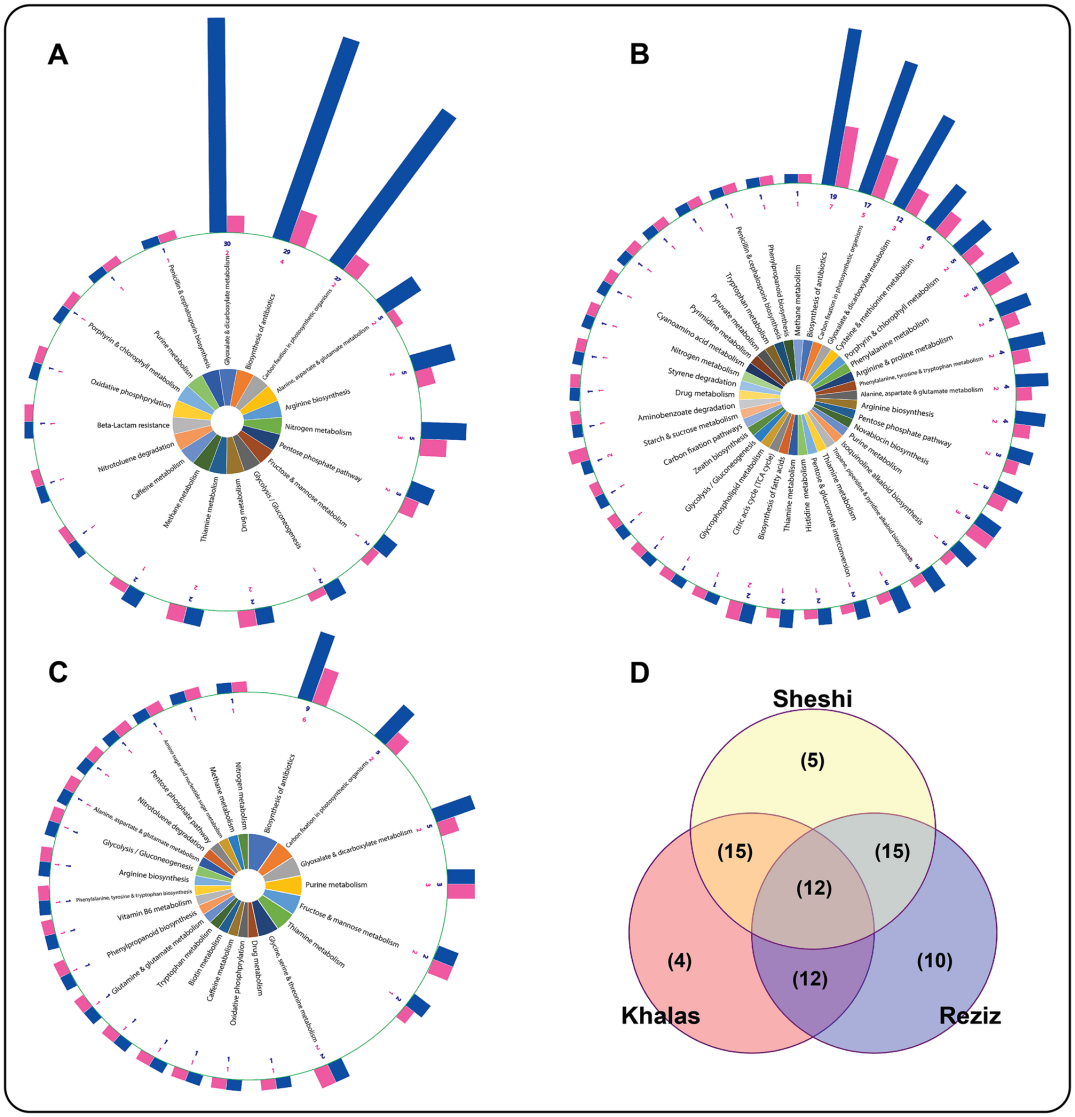
Pathways assigned to the drought-responsive ESTs in Khalas (**A**), Reziz (**B**), and Sheshi (**C**), after the elucidation of their KEGG pathways. The graph's central part illustrates the pathways' name; the outer shows the histogram, the number of enzymes (purple), and the number of genes encoding the enzymes (blue). The Venn diagram represents the number of common and unique pathways in three date palm cultivars (**D**).

The KEGG pathway analysis of each cultivar revealed that three cultivars had the highest number of ESTs related to antibiotic synthesis; 29 ESTs (encoding 4 enzymes) in Khalas (Fig. 4A), 19 ESTs (encoding 7 enzymes) in Reziz (Fig. 4B), and 9 ESTs (encoding 6 enzymes) in Sheshi (Fig. 4C). In Khalas and Reziz, the KEGG analysis mapped higher numbers of ESTs in glyoxalate and dicarboxylate metabolism (Khalas 30 ESTs and Reziz 12 ESTs), followed by carbon fixation in photosynthetic organisms (Khalas 27 ESTs and Reziz 17 ESTs, respectively) (Fig. 4).

The Venn analysis for deciphering common and unique sets of ESTs expressed in these cultivars revealed that 12 pathways were upregulated in all three cultivars to ameliorate the drought stress. Coherently, 15 pathways associated ESTs were found to be commonly upregulated in Khalas and Sheshi, as well as between Reziz and Sheshi (Fig. 4D). Whereas, Khalas and Reziz shared 12 common pathways. Besides common pathways, ESTs belonging to unique pathways were also upregulated; in Khalas 4, Reziz 10, and Sheshi 5 (Fig. 4; Table S2).

**Figure 5:**
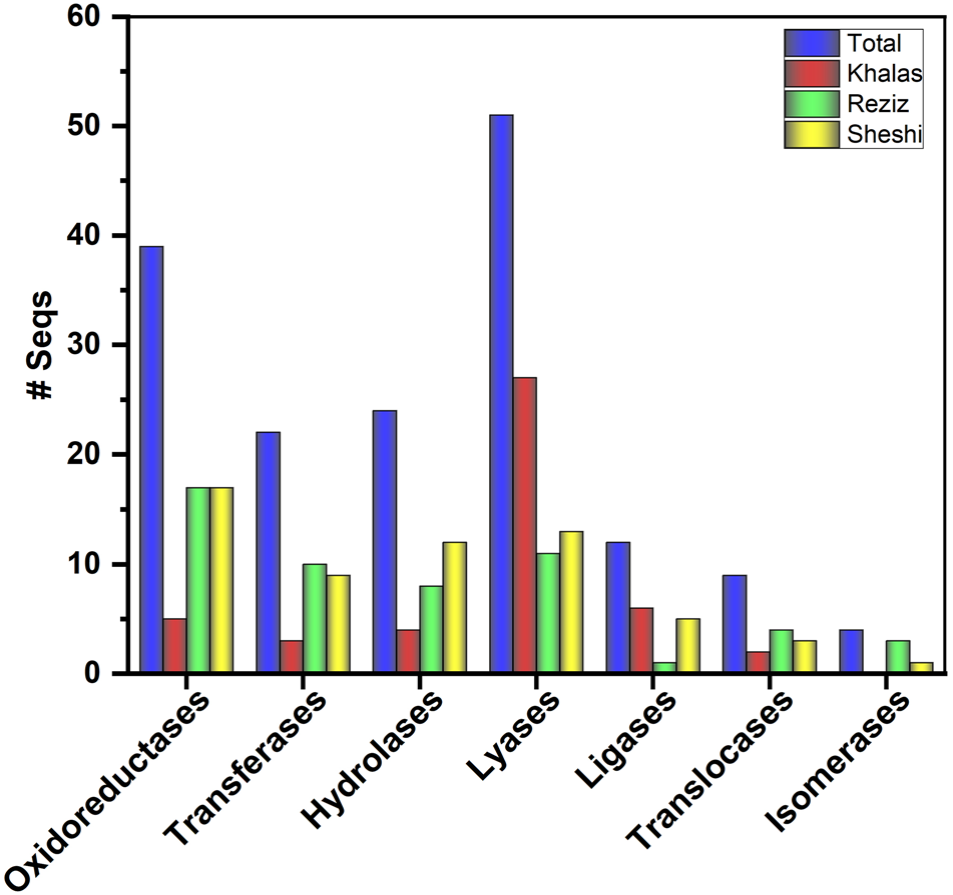
KEGG ontology (KO) distribution of major enzymes code of all 1118 drought-responsive ESTs in date palm cultivars. The total number of EC belonging to each enzyme class was mentioned in blue, while EC belonging to Khalas are mentioned in red, Reziz in green, and Sheshi in yellow.

Enzyme commission numbers are a hierarchical numbering system for enzymes based on the reaction they catalyze. EC distribution analysis revealed that 39 ESTs encode oxidoreductases (Khalas encoding 5, Reziz 17, and Sheshi 17), 22 ESTs encode transferases (Khalas encoding 3, Reziz 10, and Sheshi 9), 23 ESTs encode hydrolases (Khalas encoding 4, Reziz 7, and Sheshi 12), 51 ESTs encode lyases (Khalas encoding 26, Reziz 11, and Sheshi 14), 11 ESTs encode ligases (Khalas encoding 6, Reziz 1, and Sheshi 4), and 9 ESTs encode translocases, and 4 ESTs encode isomerases (Fig. 5). The details of the pathways, along with EC numbers for each enzyme and the locations of their coding genes are listed (Table S1).

## Discussion

The study described here is the first to examine and appraise the molecular response to drought stress of three date palm cultivars. The drought-exposed date palm plants exhibited a comparable level of phenotype to each other. However, under natural conditions, the physiological and molecular parameters, as well as expression levels of the date palm genes, can vary due to the constantly changing environmental conditions (temperature and humidity), soil capacity to retain moisture, rainfall patterns, and rate of transpiration. During any stress, young leaves and roots are mainly affected parts of the plant^33,34^, but in the case of date palm plants, the effect of drought is more pronounced on mature leaves (Munir personal communication). Date palm plant has a solitary shoot ending in a crown of leave (monopodial) without having an apparent branching system. So, date palm leaves were used in the study to quantify and appraise the drought stress at the transcriptomic level.

The findings of this study revealed that different physiological attributes were modulated to varying levels in the date palm cultivars. The cvs. Khalas and Reziz maintained better leaf RWC than cv. Sheshi. It could be because these cultivars were able to maintain better water potential and osmotic potential in their leaves and roots during drought. The overall improved water utilization system of cvs. Khalas and Reziz are linked to the plant's overall genetic makeup, which contributes to its drought-resistant physiology. Transgenic *Arabidopsis* plants overexpressing the date palm gene were previously found to have superior water management under abiotic stress conditions^35^. Moreover, as the plant triggers adaptation mechanisms to resist water stress, the increase in respiration at lower RWC would correspond to an increase in metabolism^36^.

Plants rapidly deploy a plethora of information (gene upregulation) in response to the onset of stress and orchestrate stress-responsive genes via complex regulatory networks. A study mapping the heat and drought-responsive ESTs in date palm reported similar sets of ESTs to ours^29^. Another study used 2D electrophoresis and mass spectrometry to analyze the proteome repertoire of drought and salinity exposed date palm plants. They deciphered that three common proteins, ATP synthase alpha, beta unit, and Ribulose-1,5-bisphosphate carboxylase (Rubisco), were differentially expressed under both stresses, while nine proteins, including chlorophyll A-B binding protein, superoxide dismutase, light-harvesting complex I protein, phosphoglycerate kinase, transketolase, and phosphoribulokinase, expressed to similar levels in salt stress^28^.

Our data revealed an upregulation of ESTs involved in metabolic and cellular processes, different kinds of stimuli, protein binding, biological regulations, catalytic activities, structural molecule, and transporter activities. These findings are consistent with earlier reports in date palm, where a similar set of ESTs with comparable MF, CC, and BP profiles was observed under the saline stress^37^ and normal physiological conditions^38^. Although our results demonstrated that a similar set of ESTs was upregulated, but we may speculate on two things: first, the expression level of these ESTs would be relatively high as we generated ESTs by subtracting them from control plants; and second, the molecular mechanism underlying these regulations may differ substantially. Additionally, increased expression of these ESTs may be a common strategy for coping with a variety of stressors.

The current results inferred that the upregulation of genes, inclusively and conclusively, involved in photosynthesis, ATP synthesis, maintaining cell and cell membrane integrity, oxidation–reduction pathways, maintaining homeostasis, metal ion binding proteins, and protein synthesis. Under drought conditions, photosynthesis is severely compromised. As photosynthesis sensors, chloroplasts are directly and indirectly affected by light intensity and water and moisture availability. Under drought stress, chloroplast communicates retrogradely with the nuclear genome to mediate the expression of nuclear-encoded plastid proteins to ameliorate the stress^39^ and maintain their homeostasis by regulating the activity of photosynthesis-related proteins. During drought conditions, CO_2_ uptake is reduced due to impaired ATP synthesis and metabolism^40^. Our results demonstrated elevated levels of ATP synthesis-related genes. A mitochondrial terminal oxidase, alternate oxidase, is responsible for the uncoupling of the energy consumption of ATP synthesis in *N. tabacum* plants. The overexpression of this enzyme improved the photosynthetic performance under drought conditions^41^. Nonetheless, some findings revealed that photosynthesis was compromised through stomatal apertures not by ATP synthesis^42^. Nonetheless, our results demonstrated that cvs. Khalas and Reziz used a greater proportion of photosynthetic machinery and associated parameters than cv. Sheshi to cope with drought, which corroborates previous research demonstrating a link between photosynthetic rate and intercellular CO_2_ concentration in date palm^43^. Additionally, ATP synthase was upregulated in cv. Reziz, which might be linked to its hyper ability to withstand against drought stress compared to the other two cultivars. The peptide mass fingerprint analysis of drought-exposed muskmelon revealed an upregulation of proteins involved in photosystem, nucleotide biosynthesis, DNA binding, and stress-responsive proteins^44^. Our findings corroborate to Kim et al. (2007), who demonstrated the upregulation of DNA-binding transcripts and these proteins were involved in post-transcriptional gene expression processes and in the regulation of abiotic stresses^45^. Conclusively, chloroplast plays a core role in mitigating drought stress by up-regulating the photosynthetic protein repertoire, ATP bindings and signaling pathways.

Rubisco is a rate-limiting enzyme in the Calvin cycle^46^, and its structure, level, and activity are severely compromised under drought stress^47,48^, which was linked to an inhibitor of Rubisco activity in tobacco leaves^48^. In drought susceptible wheat germplasm, the Rubisco level severely dropped in the flag leaves, resulting in early senescence, while it remained steady in the drought-tolerant germplasm^49^. A study corroborated our findings that analyzed the proteome repertoire of drought- and salt-responsive date palm plants and exhibited significant upregulation in the levels of Rubisco and its components^26,28^. So, it is speculated that elevated levels of Rubisco are a common mechanism among date palm cultivars to orchestrate normal cell functions to mitigate drought stress. By triggering the precursors of Rubisco activase and Phosphoglycerokinase, the level of rubisco and carbohydrates synthesis, including 3-phosphoglycerate, can be enhanced^50^. Subsequently, this leads to glucose and pinitol production, and both function as major osmolytes under drought conditions^51^.

Light-harvesting chlorophyll-binding a/b proteins (LHCB) are the most abundant protein in nature and are part of PS-II. Numerous ESTs encoding PS-I and PS-II components were upregulated in all three date palm cultivars. Several members of the LHCB family (LHCB1-to-LHCB6) are known in plants, where they serve as antenna complexes for light harvesting and photoprotection^52^. Disruption or down-regulation of any LHCB members led to a reduction in drought tolerance, responsiveness of stomatal movement to abscisic acid (ABA), and compromised ROS homeostasis^53^. Water stress decreases up to 55% chlorophyll contents in plants^54,55^ and in the present study, a substantial reduction in the chlorophyll contents could be attributed to the sensitivity of this pigment to escalating water stress and impaired photophosphorylation process due to decreasing leaf water potentials^56–58^. A peanut gene related to chlorophyll biosynthesis (*AhGLK1*) was up-regulated during drought stress, and its expression into glk1glk2 mutants *Arabidopsis* plants induced a green phenotype and recovery from drought stress^59^. Similarly, *Brassica napus* Drought 22 kD (BnD22), a chlorophyll-binding protein, protected younger tissues from adverse conditions and sustained sink growth under stress conditions^60^. Drought sensitivity was found to be linked with impaired LHCBs and chlorophyll contents in wheat^61^. All the previous studies concur with the findings presented here, signifying that expression of LHCBs or chlorophyll pigments or photosynthetic antenna protein complex is a common mode of action for alleviating drought stress in different plant species.

ESTs encoding monooxygenases (MO) were expressed in all three date palm cultivars, with the highest number of MOs were observed in Khalas. Flavin-containing MOs (FMOs) are most abundant in plants; 29 FMO like genes have been identified in Arabidopsis only. FMOs catalyze the transfer of hydroxyl group to small nucleophilic atoms such as iodine, nitrogen, selenium, and sulfur^62^, and function in auxin biosynthesis, osmoregulation, metabolism of glucosinolates, stabilizes photosynthetic structure, pathogen defense and abiotic stresses^62,63^. The overexpression of two Arabidopsis FMO genes, YUC6 and YUC7, enhanced drought tolerance, auxins biosynthesis, and reduced ROS levels^64,65^. The expression of choline MO in tobacco plastids catalyzed the conversion of choline to betaine aldehyde (glycine betaine [GB]), improved photosynthetic rate, and increased tolerance to drought/salt stress^66^; in addition, it up-regulated GB synthesis in the young tissues of drought-stressed plants^67^. Similarly, 3-to-5 folds increase in choline MO was observed in Sugar beet and Amaranth plants under drought conditions^68^. In *Arabidopsis* and wheat plants, MOs indirectly enhance drought and salinity tolerance by interacting and mediating the constitutive expression of methionine sulfoxide reductase^69^. The role of MOs towards drought and salinity tolerance is very versatile and accomplished by the interplay of different biochemical mechanisms. The expression of ESTs related to MOs in three date palm cultivars is suggestive of maintaining the water balance, reducing the ROS level, improving the photosynthetic rate and ultimately ameliorating the drought stress.

Metallothioneins (metals ion binding proteins [MIB]) are biological chelators that regulate metal ions homeostasis and buffer the damage caused by different abiotic stresses, including drought^30,70,71^. An upregulation of different MIBs (such as Cu-, Zn-, Fe-, Ca-ion binding) in three date palm cultivars was observed. The zinc finger proteins (ZFP) are well-studied ZIB proteins that are abundant in different plant species. About 176 ZFP have been identified in *Arabidopsis*, 126 in wheat, and 109 in *Populus trichocarpa*^72–74^. ZIBs improve abiotic stress tolerance in plants by scavenging ROS, inducing ABA, enhancing soluble sugars, and reducing water loss by closing the stomatal apertures^73,75,76^. The overexpression of a ZFP, *BBX24*, enhanced the tolerance of *Chrysanthemum morifolium* plants to drought and salt^77^. Additionally, certain ZFPs, AtTZF, AtRZFP, DST, ZAT12, interact with other proteins like *Mediator of ABA-regulated Dormancy 1* (MARD1), *Fer-Like Iron Deficiency-Induced transcription Factor* (FIT), *Jasmonate Zim protein 7* (JAZ7) and *abscisic acid receptor* (PYL5) to ameliorate salt and osmotic stress in various plants^73,76,78,79^. Under drought conditions, the *Oryza sativa metallothionein 1a* (OsMT1a) and cotton *metallothionein 3a* (GhMT3a) genes were upregulated, with the highest levels in the roots^80,81^.

Drought poses unique challenges to plants at the cell wall and the membrane levels. Plants try to circumvent water scarcity by restructuring the polysaccharide-rich cell wall and lipid bilayer cell membrane. Our data indicated that approximately 11% ESTs related to integral components of the cell membrane and around 8% ESTs related to chloroplast thylakoid membranes were expressed. Inevitably, more ESTs involved in cell structure modification, mitotic cell cycle, microtubules, and cytoskeleton were upregulated in cvs. Reziz than Khalas. This demonstrated that Reziz deployed more cellular resources to re-structure its cellular integrity than the other two cultivars. Under drought conditions, barley plants extensively rearranged actin filaments of leaf cells^82^, and maize plants produced the highest levels of chloroplast thylakoid membrane proteins^83^. Drought stress perturbed the phospholipid composition of wheat plants by affecting the phosphatidylcholine/phosphatidylethanolamine ratio and unsaturation in the fatty acyl chains^84^. Phospholipids can regulate aquaporins, the constitutive expression of the AQP gene, StPIP1, in potato plants improved drought tolerance, nonstructural carbohydrates level, and WUE^85^. Our physiological data revealed a small difference in WUE between stressed and non-stressed plants, apart from cv. Khalas, which differed substantially from the other cultivars after 90 days of stress. Our findings coincide with those of Helaly et al.^20^, who reported an increase in WUE in date palm cvs. Shamia and Amri with the increase in water stress. So, we speculated that upregulation of WUE, ESTs associated with integral membrane components, and thylakoid membranes play a pivotal role in improving drought tolerance in the date palm cultivars. ATP binding proteins (ABPs) play a critical role in adaptation to water scarcity via regulating the stomatal openings, increasing active transport of solutes, and ABA^86,87^. ABP is a large family of membrane transporters in plants with over 200 known members in rice, *Lotus japonicus,* and *Arabidopsis* plants. The *Arabidopsis* ABP, AtABCG25/WBC26 transporter, is an ABA efflux carrier, and its overexpression reduced the water loss by facilitating ABA delivery to guard cells^88^; similarly, expression of AtABCG36 in transgenic *Arabidopsis* plants contributed to drought stress and salt resistance^89^. An increased level of ABP and GBP was observed in *Arabidopsis* and *Betula halophila* plants under drought stress, and the significant increase in ABP is consistent with the increased activity of ATPase^90,91^. Thus, all three date palm cultivars opted for similar molecular mechanisms to mitigate the drought stress by overexpressing the ABP-associated ESTs.

Besides, several other ESTs having roles in metabolic processes, including protein modification, regulation of RNA metabolic processes, and purine ribonucleotide/nucleoside binding proteins, were also identified. Similar enriched gene networks have also been deciphered in *Dendrobium wangliangii*, an endangered species due to climate change^92^. In general, the response of the cv. Sheshi to drought was comparable to that of the cv. Reziz, as similar sets of drought-responsive genes were transcribed in both cultivars (Fig. 5). Nonetheless, a greater number of expressed ESTs were related to the proteolysis process. Hieng and his co-workers showed that hyper-expression of proteases is an essential part of abiotic stress tolerance in *Phaseolus vulgaris*, especially drought tolerance^93^, and an enhanced level of cysteine protease and increased proteolytic activity was a hallmark of drought tolerance in wheat leaves^94^.

The KEGG pathways results demonstrated that date palm ESTs were related to over 50 pathways. The KEGG pathway revealed that three cultivars had the highest number of ESTs related to antibiotic synthesis. Similar findings have been reported earlier in date palm, where the highest numbers of enzymes were found associated with antibiotics synthesis^95^. In Khalas and Reziz, the KEGG analysis mapped higher numbers of ESTs in glyoxalate and dicarboxylate metabolism. Both of these two pathways have a pivotal role in the amelioration of drought- and abiotic stresses^96–98^. Drought-induced SSH library of upland cotton was studied by KEGG pathway, and approximately 12% of ESTs were found related to glyoxylate and dicarboxylate metabolism^99^. The certain glyoxylate and dicarboxylate-related proteins in *B. napus* plants decreased under cold stress, indicating their role against abiotic stress^100^. In salt-stressed tomato plants, 6 carbohydrate metabolic pathways, including glyoxylate and dicarboxylate metabolism, played a significant role in adaptation to salt stress^101^. Contrasting to these findings, a down-regulation of glyoxylate and dicarboxylate metabolism was demonstrated in drought-induced *Eruca vesicaria*^102^, Tree peony^103^, and sesame plants^104^. A precise reason of this is difficult to rationalize and far from a conclusion and needs further analysis. The date palm plants were countering the drought stress by improving the chlorophyll biosynthesis by activating the key enzymes in porphyrin and chlorophyll metabolism. The upregulation of carbon fixation, glyoxylate and dicarboxylate metabolism, and enzymes involved in chlorophyll metabolism under implied drought conditions, revealed their majestic role to neutralize the drought stress.

Enzyme Commission (EC) numbers distribution is a hierarchical numbering system for enzymes based on the reaction they catalyze. We performed the EC distribution analysis and found different ESTs belonging to all the major classes of enzymes. Similar findings have also been reported earlier in date palm cv. Sukary^38^.

Although we were able to map a decent number of ESTs against the available repositories, representing ~ 50% of the ESTs collection, nonetheless, a huge number of ESTs were novel and could not be mapped, as gene annotation data is not available. The role of novel ESTs in date palm drought tolerance would be substantially important and could interest future studies. In addition, such genes could provide novel clues and can be used to develop drought (or abiotic stresses) tolerant date palm plants.

## Materials and methods

### Experimental site, plant material and growth conditions

The present study was conducted during the years 2018–2019 at the Date Palm Biotechnology department, Date Palm Research Center of Excellence, King Faisal University, Saudi Arabia (Latitude 25° 16′ 17.8896″ N, Longitude 49° 42′ 22.446″ E). Five-year-old, uniform, mature offshoots of commercial date palm cvs. Khalas, Reziz and Sheshi, raised through tissue-culture were obtained by Balakrishnan Sudhakar from SAPAD Tissue Culture Date Palm Co., Dammam, Saudi Arabia. The formal identification of the date palm cultivars was performed by M. Munir (Plant physiologist). These plants, however, were not deposited in a publicly accessible herbarium because they all were commercial cultivars. Plants were then potted (80 L, 61 cm diameter, 46 cm height) in a 1:2 mixture of sandy loam soil (Aridisol soil) and sphagnum peat moss compost.

The sandy loam soil was collected from the top layer (0–20 cm) of a nearby barren field with no sign of vegetation (Latitude 25° 16′ 27.4224″ N and Longitude 49° 42′ 24.8328″ E). It was air-dried at room temperature, sieved through 2 mm stainless steel wire mesh, and sterilized in an autoclave at 121 °C for 2 h^105^. Sandy loam soil contained 79.94% sand, 8.13% silt, 11.93% clay, 7.42 pH, 3.87 dS m^−1^ EC, 3.57% moisture content, 1.57 g cm^−3^ bulk density, 0.24% organic matter, 17.68 mg kg^−1^ total nitrogen, 11.18 mg kg^−1^ phosphorus, 13.58 mg kg^−1^ potassium, and 20.53 mg kg^−1^ sodium. The sphagnum peat moss had 5.2 pH, 2.3 dS m^−1^ EC, 39.73% moisture content, 0.94 g cm^−3^ bulk density, 92.47% organic matter, 976 mg kg^−1^ total nitrogen, 341 mg kg^−1^ phosphorus, 543 mg kg^−1^ potassium, and 283 mg kg^−1^ sodium. Prior to drought induction, the date palm plants were acclimatized in a sterilized growth room for 3 months. The mean diurnal temperature of the growth room was 27 ± 2 °C, relative humidity 60%, and 14 h day^−1^ photoperiod (800 µmol m^−2^ s^−1^ photosynthetic photon flux density—PPFD). Three replicated control plants of each cultivar were watered at 100% field capacity (FC), while a gradual and progressive water deficit plan was followed to induce drought stress at 25% FC in three plants of each cultivar^106^. The experiment was laid out on a completely randomized design with three replications of each date palm cultivar.

### Relative water content

The estimation of relative water contents (RWC) of leaves was accomplished in accordance with Cao et al.^107^ after 90 days of water stress. Three fully expanded leaves were excised from the second whorl of the plant of each cultivar, and their fresh weight was recorded within 2 h. Leaf turgid weight was obtained after immersing these leaves for 16 h in distilled water. After soaking, leaves were carefully dried with tissue paper prior to the determination of turgid weight. Leaf dry weight was obtained after drying the leaves sample for 72 h at 70 °C. Relative water content was calculated from the following equation:

$$RWC=\frac{\text{Leaf fresh weight }{-}\text{ Leaf dry weight}}{\text{Leaf turgid weight }{-}\text{ Leaf dry weight}} \times 100$$

### Estimation of physiological parameters

Among physiological parameters, leaf chlorophyll content was calculated after 90 days of water stress using a chlorophyll meter (SPAD 502, Konica–Minolta, Japan). The photosynthesis (*Pn*), stomatal conductance (*gs*), transpiration (*E*), and intercellular CO_2_ concentration (*Ci*) were estimated at 0, 45, and 90 days of water stress using the Li-6400XT photosynthesis system (LiCor Inc., Lincoln, NE, USA) as described previously^108^. A leaf area of 6 cm^2^ was inserted in the Infra-Red Gas Analyzer (IRGA) chamber for the estimation of photosynthesis and related parameters. The leaf temperature of the IRGA chamber was adjusted at 25 °C. Inside the chamber, the reference and sample CO_2_ were set at 400 µmol m^2^ s^−1^ with 500 µmol s^−1^ airflow. The photosynthetic WUE was calculated by *Pn*/*E* after 0, 45, and 90 days of drought induction.

### Sample collection and total RNA isolation

Two grams (g) of mature leaf tissues (middle leaflets) were collected at fortnight intervals throughout the year, snap-frozen in liquid nitrogen, pooled, and stored at − 80 °C until used. To isolate total RNA from frozen samples, they were ground in a pestle with mortar. A total of 72 samples were subjected to total RNA extraction using a unique and modified protocol. Ten ml of lysis buffer (2% CTAB, 100 mM EDTA, 0.5% SDS) was added and mixed to the ground tissues, followed by 5 ml of ice-cold chloroform and 2 ml of water-saturated phenol and isoamyl alcohol (2:1). After mixing thoroughly, the homogenate was transferred to a sterile 50 ml polypropylene (PP) tube and incubated in a water bath at 40 °C with intermittent mixing. The resultant lysate was spun at 20,000 rpm at 4 °C for 10 min. The supernatant was carefully aspirated into a nuclease-free 50 ml centrifuge tube and mixed with an equal volume of phenol:chloroform mixture (1:1). The mixture was incubated in a rocker for 30 min at 10 rpm, then centrifuged at 20,000 rpm. The supernatant was transferred to a nuclease-free PP tube. Muscle glycogen (5 µg ml^−1^) and 1/5 volume of 3 M sodium acetate were added to the supernatant and inverted gently for 6‒8 times. Two volumes of chilled isopropanol were added, mixed, and incubated overnight at −80 °C. The next morning, the total RNA was harvested by centrifuging the contents at 20,000 rpm for 30 min at 4 °C. The resultant RNA palette was air-dried and then re-suspended in 10 mM TE buffer. To get rid of any DNA contamination, the extracted total RNA solution was first treated with DNase I (1 U 100 μL^‒1^) and then phenol:chloroform (1:1) extraction was performed. Finally, the total RNA was precipitated at 20,000 rpm for 20 min and the resultant pellet was air-dried for two minutes and dissolved with nuclease-free water. The isolated RNAs' quality and quantity were assessed spectrophotometrically (2100 Bioanalyzer, Agilent, USA) and electrophoretically. All plasticware and utensils used in RNA extractions were pretreated with 0.8% of the Diethyl Pyrocarbonate (DEPC) solution.

### Construction of cDNA and suppressive subtractive hybridization (SSH)

Messenger RNA (mRNA) was isolated from their respective purified total RNA using the mRNA purification kit (Poly A purist MAG kit, Thermo Fisher Scientific, Massachusetts, USA). The synthesis of cDNA, both first and second strands, was carried out using the SMARTer cDNA synthesis kit (Takara Bio Inc., California, USA).

The drought-responsive SSH library of expressed transcripts of each cultivar was generated by two-step subtraction hybridization using Clontech^®^ PCR-Select™ cDNA Subtraction Kit (Takara Bio Inc., California, USA). The cDNA of drought-stressed date palm (tester) plants was hybridized with the cDNA of the control date palm (driver) plants to subtract the cDNA of the driver plants. The remaining unhybridized cDNA, representing differentially expressed genes, were subjected to second-strand synthesis and PCR amplification. The resulting double-stranded (ds) cDNAs were ligated into a TOPO-TA cloning vector (Thermo Fisher Scientific, Massachusetts, USA), transformed into *Escherichia coli* (strain DH5α), and spread on an Ampicillin and IPTG/X-Gal LB agar plates. Colonies with successful insertion were cultured to isolate the recombinant plasmids (GeneJET Plasmid Miniprep, Thermo Fisher Scientific, Massachusetts, USA) and then sequenced to make drought-specific libraries of cvs. Khalas, Reziz and Sheshi. In the end, a unique identifier nomenclature (DPRC2K16C 1-2733) was developed to assign a unique number to each clone.

### ESTs sequencing and assembly

Purified clones were sequenced with M13 (‒20) forward sequencing primers using the Sanger sequencing platform. The obtained sequences were cleaned by trimming vector and adapter sequences using online sources (www.ncbi.nlm.nih.gov). The finally cleaned ESTs were BLASTx searched (www.ncbi.nlm.nih.gov)^109^ using a non-redundant (nr) database with an acceptable cutoff E-value at ≤ 1.0E-−05. If no homology was found in the BLASTx search, then the BLASTn algorithm was used in conjunction with the nucleotide sequence database with an acceptable cutoff E-value at ≤ 1.0E−05. Besides, the reference data were used from the European Molecular Biology Laboratory (EMBL) and the DNA Data Bank of Japan (DDBJ).

### Annotation of the ESTs

To annotate and map the date palm ESTs, OmicsBox (v1.2.4) was used^110^, and the functional description of different plant species was followed to assign the functions. Additionally, InterProScan^111^, gene mapping, and gene ontology (GO) tools were used for an additional level of functional annotations and assigning pathways. Furthermore, the results of InterProScan were merged with GO annotation to yield more precise data. Initially, the drought-responsive date palm ESTs were annotated and considered credible according to the BLASTx results against the non-redundant (nr) database with an E-values ≤ 0.05. However, this ends up with just a small number of ESTs with GO analysis. Subsequently, the analyses were carried out at E-value 10 to yield maximum GO data. The unmatched ESTs were further searched through the Pfam database in the OmicsBox. For the ESTs with multiple BLAST hits, only the highest score hit was considered credible and included in the analysis. In addition, enzyme mapping of ESTs was performed using OmicsBox and KEGG pathways were elucidated using KEGG orthologs (KO) using the OmicsBox default parameters.

### Statistical analysis

The recorded data were analyzed using Genstat software, 14th Edition (VSNi, Hemel Hempstead, England). Duncan’s Multiple Range Test (DMRT) was applied to determine the least significant difference between treatment means (*p* ≤ 0.05), if the ANOVA showed significant differences at a 5% probability level.

### Ethical approval

All the experimental protocols involving plants adhered to relevant ethical parameters/regulations and were approved by an indigenous institutional committee.

## Conclusions

Our study examined the response of three date palm cultivars (Khalas, Reziz, and Sheshi) to drought stress at physiological and transcriptome levels. Although drought stress significantly affected leaf RWC, chlorophyll content, photosynthesis system, and WUE, however, date palm cvs. Khalas and Reziz thrive well under the stressed environment (at 25% FC) compared to cv. Sheshi. At the transcriptomic level, each date palm cultivar deployed not only common but also distinct mechanisms for drought stress amelioration, indicating their indigenous potential to ameliorate drought stress. The response of cvs. Khalas and Sheshi to drought amelioration was comparable, while cv. Reziz deployed more pathways associated ESTs, showing its higher potential to withstand against drought conditions. A large number of ESTs were novel and indigenous to date palm, and their functions could not be assigned due to a lack of nucleotide sequence homology with other plant species. However, this could be an area of interest for future studies. To the best of our knowledge, this is the first study in which the response of three elite date palm cultivars at the molecular level was studied and appraised.

## Supplementary Information

Supplementary Information.

## Data Availability

All the data related to this study is contained in this manuscript and supplementary files. The ESTs sequence data has been submitted to the databank, comprising 344 from Khalas (Bioproject PRJDB11270; accession # HX998187-HX998530), 387 from Reziz (Bioproject PRJDB11271 accession # HX998531-HX998917), and 377 from Sheshi (Bioproject PRJDB11272; accession # HX998918-HX999293).
