# Supplementary material for: Common and novel metabolic pathways related ESTs were upregulated in three date palm cultivars to ameliorate drought stress
Source: Sci Rep. 2022 Sep 2;12:15027. doi: 10.1038/s41598-022-19399-8 (PMC9440037; doi:10.1038/s41598-022-19399-8)
Supplement: Supplementary file 1 — Supplementary Information. [file 41598_2022_19399_MOESM1_ESM.docx]

Supplementary Material

Common and novel metabolic pathways related ESTs were upregulated in three date palm cultivars to ameliorate drought stress

Mohammed Refdan Al-Hajhoj^1^, Muhammad Munir^2^, Balakrishnan Sudhakar^2^, Hassan Muzzamil Ali-Dinar^2^, Zafar Iqbal^3*^

^1^ Department of Arid Land Agriculture, College of Agriculture and Food Sciences, King Faisal University, PO Box 31982, Al-Ahsa, Saudi Arabia 1; malhajhoj@kfu.edu.sa

^2^ Date Palm Research Center of Excellence, King Faisal University, PO Box 31982, Al-Ahsa, Saudi Arabia on 2;

M. Munir – mmunir@kfu.edu.sa

B. Sudhakar - meetsudhakar@gmail.com

H. M. Ali Dinar – hdinar@kfu.edu.sa

***** Central Laboratories, King Faisal University, PO Box 31982, Al-Ahsa, Saudi Arabia Correspondence: zafar@kfu.edu.sa; Tel.: +966580776536


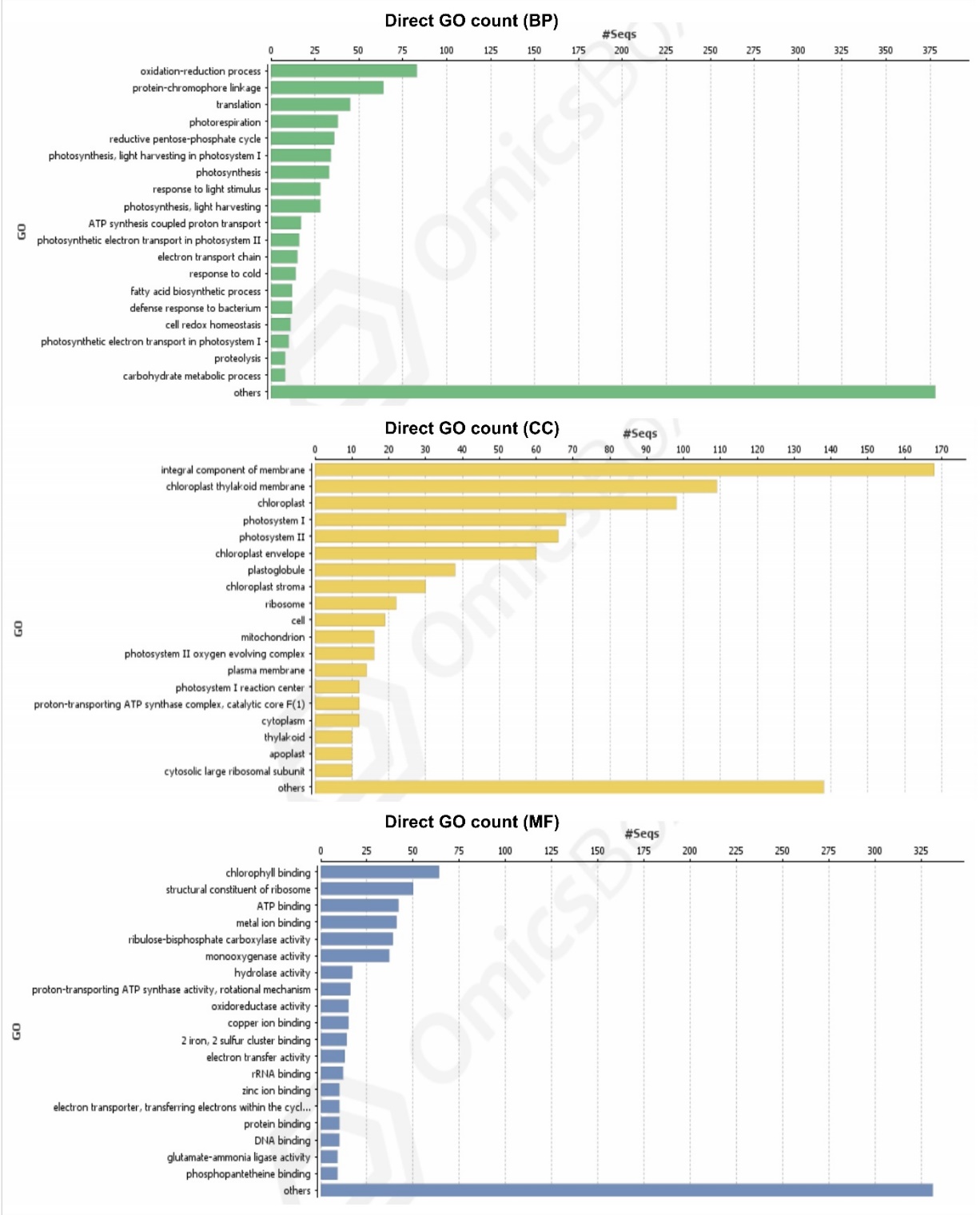


**Figure S1.** Blast2Go annotation of all the date palm ESTs based on high-score non-redundant BLASTx homology. GO classified a total of 1118 ESTs into biological process (BP), by cellular component (CC), and by molecular functions (MF).

**
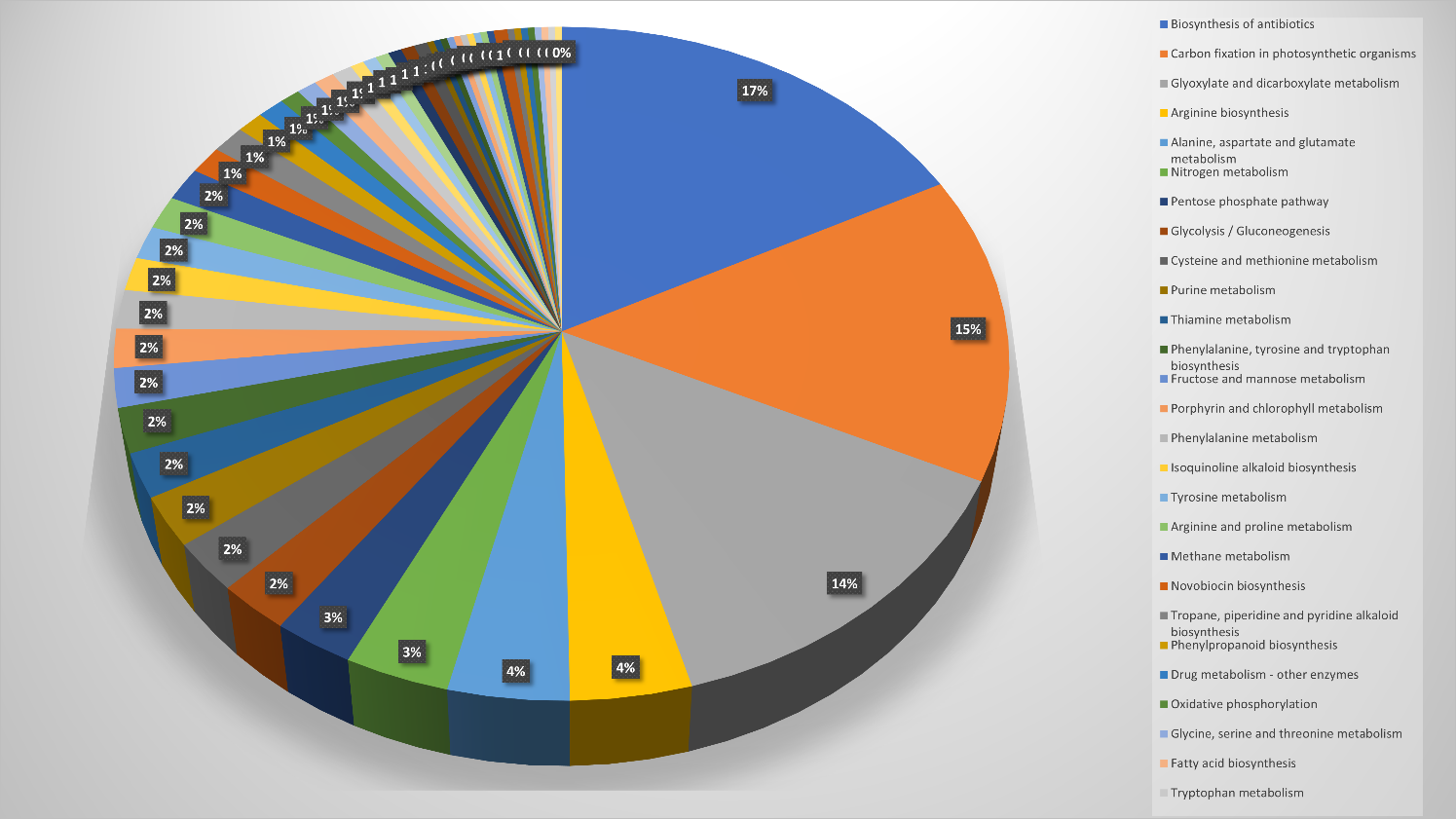
**

**Figure S2.** KEGG ontology (KO) categories of drought-responsive ESTs of all the date palm cultivars. The graph illustrates the percentage of ESTs related to a biochemical pathway.


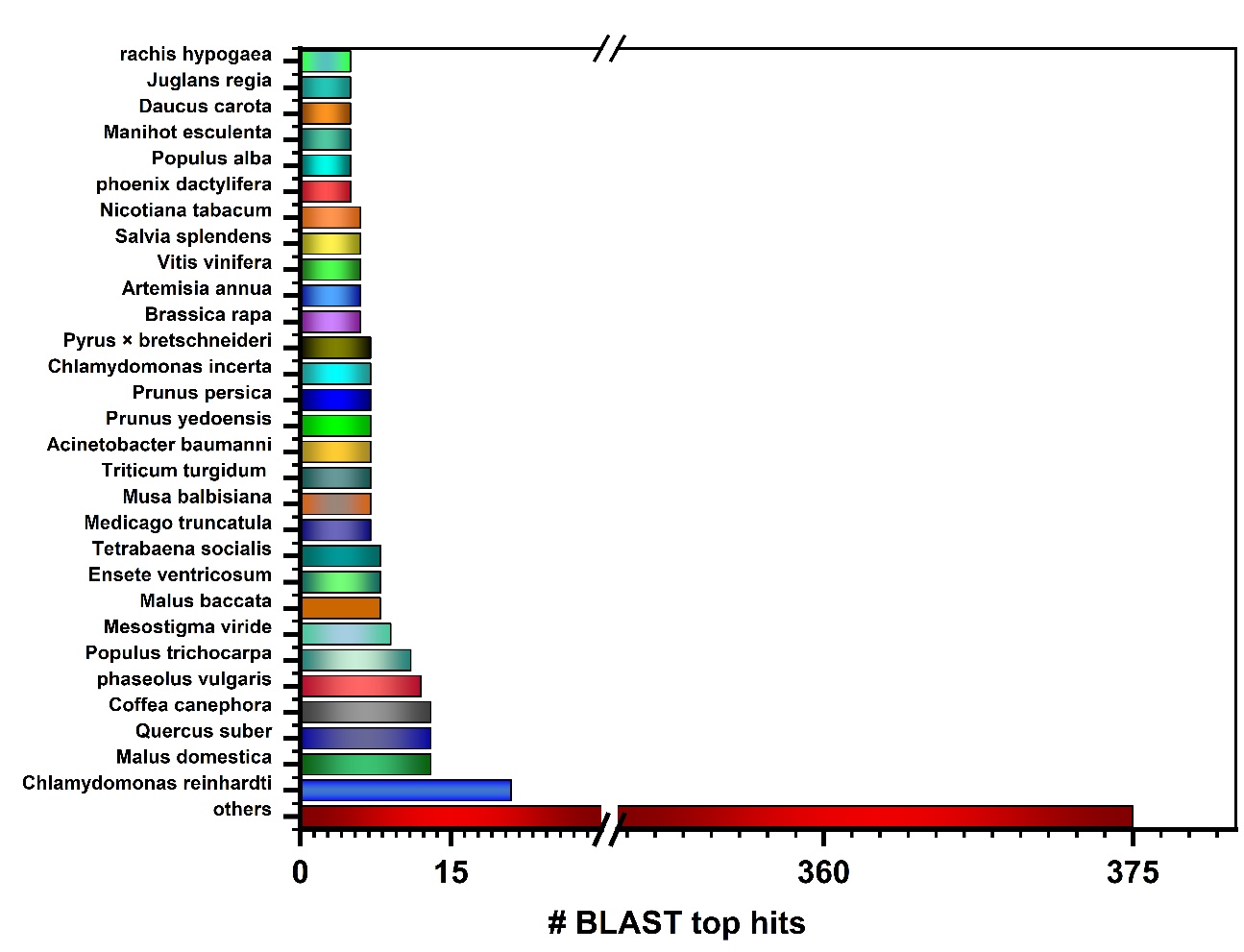


**Figure S3.** List of species showing BLAST search homology of drought-responsive ESTs of all the date palm cultivars. The graph illustrates the number of ESTs related to a species.

**Table S 1.** The details of all the ESTs related to their elucidated KEGG pathways. The list of pathway name, pathways ID, number enzyme in pathway, enzyme code and their names.

| **Pathway Name** | **Pathway ID** | **#Enzymes in Pathway** | **Enzyme and their codes** |
| --- | --- | --- | --- |
| Glyoxylate and dicarboxylate metabolism | map00630 | 4 | ec:1.1.1.37 - dehydrogenase, ec:4.1.1.39 - carboxylase, ec:6.3.1.2 - synthetase, ec:1.11.1.6 - equilase |
| Glycolysis / Gluconeogenesis | map00010 | 6 | ec:2.7.2.3 - kinase, ec:3.2.1.86 - phospho-beta-glucosidase A, ec:4.1.2.13 - aldolase, ec:1.2.1.59 - dehydrogenase (NAD(P)+) (phosphorylating), ec:1.2.1.12 - dehydrogenase (phosphorylating), ec:3.1.3.11 - hexose diphosphatase |
| Phenylpropanoid biosynthesis | map00940 | 2 | ec:3.2.1.21 - gentiobiase, ec:1.11.1.7 - lactoperoxidase |
| Glutathione metabolism | map00480 | 1 | ec:6.3.2.2 - ligase |
| Tryptophan metabolism | map00380 | 2 | ec:3.5.1.4 - acylamidase, ec:1.11.1.6 - equilase |
| Pyruvate metabolism | map00620 | 1 | ec:1.1.1.37 - dehydrogenase |
| Biotin metabolism | map00780 | 1 | ec:6.3.4.15 - carboxyl-carrier protein] ligase |
| Phenylalanine, tyrosine and tryptophan biosynthesis | map00400 | 3 | ec:4.2.3.5 - synthase, ec:2.6.1.1 - transaminase, ec:4.2.1.20 - synthase |
| Betalain biosynthesis | map00965 | 1 | ec:1.14.18.1 - monophenol monooxygenase |
| Cyanoamino acid metabolism | map00460 | 1 | ec:3.2.1.21 - gentiobiase |
| Caffeine metabolism | map00232 | 1 | ec:2.3.1.5 - N-acetyltransferase |
| Biosynthesis of antibiotics | map01130 | 19 | ec:2.7.2.3 - kinase, ec:2.7.4.6 - kinase, ec:4.2.3.5 - synthase, ec:1.1.1.37 - dehydrogenase, ec:5.3.1.6 - isomerase, ec:4.6.1.12 - 2,4-cyclodiphosphate synthase, ec:5.1.3.1 - 3-epimerase, ec:4.1.1.39 - carboxylase, ec:4.1.2.13 - aldolase, ec:3.5.2.6 - penicillinase, ec:6.3.5.3 - synthase, ec:1.2.1.59 - dehydrogenase (NAD(P)+) (phosphorylating), ec:2.6.1.1 - transaminase, ec:1.2.1.12 - dehydrogenase (phosphorylating), ec:5.3.1.8 - isomerase, ec:3.1.3.11 - hexose diphosphatase, ec:3.1.1.31 - phosphogluconolactonase, ec:4.2.1.20 - synthase, ec:1.11.1.6 - equilase |
| Purine metabolism | map00230 | 4 | ec:3.6.1.15 - phosphatase, ec:2.7.4.6 - kinase, ec:3.6.1.3 - adenylpyrophosphatase, ec:6.3.5.3 - synthase |
| Vitamin B6 metabolism | map00750 | 1 | ec:4.2.3.1 - synthase |
| Glycerolipid metabolism | map00561 | 1 | ec:3.2.1.22 - melibiase |
| Styrene degradation | map00643 | 1 | ec:3.5.1.4 - acylamidase |
| Arginine biosynthesis | map00220 | 3 | ec:6.3.1.2 - synthetase, ec:2.6.1.1 - transaminase, ec:3.5.1.2 - glutaminase I |
| Nitrotoluene degradation | map00633 | 1 | ec:2.3.1.5 - N-acetyltransferase |
| Galactose metabolism | map00052 | 1 | ec:3.2.1.22 - melibiase |
| Amino sugar and nucleotide sugar metabolism | map00520 | 1 | ec:5.3.1.8 - isomerase |
| Methane metabolism | map00680 | 3 | ec:1.1.1.37 - dehydrogenase, ec:4.1.2.13 - aldolase, ec:3.1.3.11 - hexose diphosphatase |
| Phenylalanine metabolism | map00360 | 3 | ec:3.5.1.4 - acylamidase, ec:3.7.1.14 - hydrolase, ec:2.6.1.1 - transaminase |
| Zeatin biosynthesis | map00908 | 1 | ec:2.5.1.75 - dimethylallyltransferase |
| Sphingolipid metabolism | map00600 | 1 | ec:3.2.1.22 - melibiase |
| Nitrogen metabolism | map00910 | 2 | ec:4.2.1.1 - anhydrase, ec:6.3.1.2 - synthetase |
| Penicillin and cephalosporin biosynthesis | map00311 | 1 | ec:3.5.2.6 - penicillinase |
| Pentose and glucuronate interconversions | map00040 | 1 | ec:5.1.3.1 - 3-epimerase |
| Tyrosine metabolism | map00350 | 2 | ec:1.14.18.1 - monophenol monooxygenase, ec:2.6.1.1 - transaminase |
| D-Glutamine and D-glutamate metabolism | map00471 | 1 | ec:3.5.1.2 - glutaminase I |
| Aminobenzoate degradation | map00627 | 1 | ec:3.5.1.4 - acylamidase |
| Terpenoid backbone biosynthesis | map00900 | 1 | ec:4.6.1.12 - 2,4-cyclodiphosphate synthase |
| Novobiocin biosynthesis | map00401 | 1 | ec:2.6.1.1 - transaminase |
| Butanoate metabolism | map00650 | 2 | ec:1.1.1.304 - reductase [(S)-acetoin forming], ec:1.1.1.303 - reductase [(R)-acetoin forming] |
| Histidine metabolism | map00340 | 1 | ec:1.1.1.23 - dehydrogenase |
| Tropane, piperidine and pyridine alkaloid biosynthesis | map00960 | 1 | ec:2.6.1.1 - transaminase |
| Oxidative phosphorylation | map00190 | 2 | ec:1.6.99.3 - dehydrogenase, ec:7.1.1.2 - reductase (H+-translocating) |
| Glycerophospholipid metabolism | map00564 | 1 | ec:2.7.8.24 - synthase |
| Thiamine metabolism | map00730 | 2 | ec:3.6.1.15 - phosphatase, ec:2.8.1.7 - desulfurase |
| Cysteine and methionine metabolism | map00270 | 4 | ec:1.1.1.37 - dehydrogenase, ec:6.3.2.2 - ligase, ec:2.5.1.6 - adenosyltransferase, ec:2.6.1.1 - transaminase |
| Carbon fixation pathways in prokaryotes | map00720 | 1 | ec:1.1.1.37 - dehydrogenase |
| Glycine, serine and threonine metabolism | map00260 | 2 | ec:4.2.3.1 - synthase, ec:4.2.1.20 - synthase |
| Starch and sucrose metabolism | map00500 | 3 | ec:2.4.1.11 - synthase, ec:3.2.1.21 - gentiobiase, ec:3.2.1.86 - phospho-beta-glucosidase A |
| Drug metabolism - other enzymes | map00983 | 3 | ec:2.7.4.6 - kinase, ec:3.1.1.1 - ali-esterase, ec:2.3.1.5 - N-acetyltransferase |
| Carbon fixation in photosynthetic organisms | map00710 | 12 | ec:2.7.2.3 - kinase, ec:1.1.1.37 - dehydrogenase, ec:3.1.3.37 - SBPase, ec:5.3.1.6 - isomerase, ec:5.1.3.1 - 3-epimerase, ec:4.1.1.39 - carboxylase, ec:1.2.1.13 - dehydrogenase (NADP+) (phosphorylating), ec:4.1.2.13 - aldolase, ec:1.2.1.59 - dehydrogenase (NAD(P)+) (phosphorylating), ec:2.6.1.1 - transaminase, ec:1.2.1.12 - dehydrogenase (phosphorylating), ec:3.1.3.11 - hexose diphosphatase |
| Alanine, aspartate and glutamate metabolism | map00250 | 3 | ec:6.3.1.2 - synthetase, ec:2.6.1.1 - transaminase, ec:3.5.1.2 - glutaminase I |
| Fatty acid biosynthesis | map00061 | 3 | ec:2.3.1.85 - synthase system, ec:3.1.2.14 - hydrolase, ec:1.3.1.9 - reductase (NADH) |
| Glycosphingolipid biosynthesis - globo and isoglobo series | map00603 | 1 | ec:3.2.1.22 - melibiase |
| beta-Lactam resistance | map01501 | 1 | ec:3.5.2.6 - penicillinase |
| Pentose phosphate pathway | map00030 | 6 | ec:5.3.1.6 - isomerase, ec:5.1.3.1 - 3-epimerase, ec:4.1.2.13 - aldolase, ec:2.7.1.15 - deoxyribokinase, ec:3.1.3.11 - hexose diphosphatase, ec:3.1.1.31 - phosphogluconolactonase |
| Pyrimidine metabolism | map00240 | 1 | ec:2.7.4.6 - kinase |
| Porphyrin and chlorophyll metabolism | map00860 | 2 | ec:1.16.3.1 - ceruloplasmin, ec:1.3.3.3 - oxidase |
| Fructose and mannose metabolism | map00051 | 4 | ec:5.3.1.6 - isomerase, ec:4.1.2.13 - aldolase, ec:5.3.1.8 - isomerase, ec:3.1.3.11 - hexose diphosphatase |
| Isoquinoline alkaloid biosynthesis | map00950 | 2 | ec:1.14.18.1 - monophenol monooxygenase, ec:2.6.1.1 - transaminase |
| Arginine and proline metabolism | map00330 | 2 | ec:3.5.1.4 - acylamidase, ec:2.6.1.1 - transaminase |
| Citrate cycle (TCA cycle) | map00020 | 1 | ec:1.1.1.37 - dehydrogenase |

**Table S2.** Unique and common pathways elucidated based on KEGG pathways among and between all three date palm cultivars

| **Unique pathways** | | | **Common pathways** | | | |
| --- | --- | --- | --- | --- | --- | --- |
| **Khalas** | **Reziz** | **Sheshi** | **Khalas-Reziz** | **Khals-Sheshi** | **Reziz-Sheshi** | **Khalas-Reziz-Sheshi** |
| beta-Lactam resistance | Aminobenzoate degradation | Amino sugar and nucleotide sugar metabolism | Glyoxylate and dicarboxylate metabolism | Caffeine metabolism | Phenylpropanoid biosynthesis | Glyoxylate and dicarboxylate metabolism |
| Penicillin and cephalosporin biosynthesis | Carbon fixation pathways in prokaryotes | Biotin metabolism | Biosynthesis of antibiotics | Nitrotoluene degradation | Tryptophan metabolism | Biosynthesis of antibiotics |
| Porphyrin and chlorophyll metabolism | Citrate cycle (TCA cycle) | D-Glutamine and D-glutamate metabolism | Carbon fixation in photosynthetic organisms | Oxidative phosphorylation | Glyoxylate and dicarboxylate metabolism | Carbon fixation in photosynthetic organisms |
| Purine metabolism | Cyanoamino acid metabolism | Phenylalanine, tyrosine and tryptophan biosynthesis | Alanine, aspartate and glutamate metabolism | Glyoxylate and dicarboxylate metabolism | Biosynthesis of antibiotics | Alanine, aspartate and glutamate metabolism |
|  | Glycerophospholipid metabolism | Vitamin B6 metabolism | Arginine biosynthesis | Biosynthesis of antibiotics | Carbon fixation in photosynthetic organisms | Arginine biosynthesis |
|  | Pyrimidine metabolism |  | Drug metabolism - other enzymes | Carbon fixation in photosynthetic organisms | Alanine, aspartate and glutamate metabolism | Drug metabolism - other enzymes |
|  | Pyruvate metabolism |  | Fructose and mannose metabolism | Alanine, aspartate and glutamate metabolism | Arginine biosynthesis | Fructose and mannose metabolism |
|  | Starch and sucrose metabolism |  | Glycolysis / Gluconeogenesis | Arginine biosynthesis | Drug metabolism - other enzymes | Glycolysis / Gluconeogenesis |
|  | Styrene degradation |  | Methane metabolism | Drug metabolism - other enzymes | Fructose and mannose metabolism | Methane metabolism |
|  | Zeatin biosynthesis |  | Nitrogen metabolism | Fructose and mannose metabolism | Glycolysis / Gluconeogenesis | Nitrogen metabolism |
|  |  |  | Pentose phosphate pathway | Glycolysis / Gluconeogenesis | Methane metabolism | Pentose phosphate pathway |
|  |  |  | Purine metabolism metabolism | Methane metabolism | Nitrogen metabolism | Thiamine metabolism |
|  |  |  |  | Nitrogen metabolism | Pentose phosphate pathway |  |
|  |  |  |  | Pentose phosphate pathway | Thiamine metabolism |  |
|  |  |  |  | Thiamine metabolism | Purine metabolism |  |
